# A Dynamic Energy Budget (DEB) Model for the Keystone Predator *Pisaster ochraceus*

**DOI:** 10.1371/journal.pone.0104658

**Published:** 2014-08-28

**Authors:** Cristián J. Monaco, David S. Wethey, Brian Helmuth

**Affiliations:** 1 Department of Biological Sciences, University of South Carolina, Columbia, South Carolina, United States of America; 2 Marine Science Center, Northeastern University, Nahant, Massachusetts, United States of America; The University of Adelaide, Australia

## Abstract

We present a Dynamic Energy Budget (DEB) model for the quintessential keystone predator, the rocky-intertidal sea star *Pisaster ochraceus*. Based on first principles, DEB theory is used to illuminate underlying physiological processes (maintenance, growth, development, and reproduction), thus providing a framework to predict individual-level responses to environmental change. We parameterized the model for *P. ochraceus* using both data from the literature and experiments conducted specifically for the DEB framework. We devoted special attention to the model’s capacity to (1) describe growth trajectories at different life-stages, including pelagic larval and post-metamorphic phases, (2) simulate shrinkage when prey availability is insufficient to meet maintenance requirements, and (3) deal with the combined effects of changing body temperature and food supply. We further validated the model using an independent growth data set. Using standard statistics to compare model outputs with real data (e.g. Mean Absolute Percent Error, MAPE) we demonstrated that the model is capable of tracking *P. ochraceus*’ growth in length at different life-stages (larvae: MAPE = 12.27%; post-metamorphic, MAPE = 9.22%), as well as quantifying reproductive output index. However, the model’s skill dropped when trying to predict changes in body mass (MAPE = 24.59%), potentially because of the challenge of precisely anticipating spawning events. Interestingly, the model revealed that *P. ochraceus* reserves contribute little to total biomass, suggesting that animals draw energy from structure when food is limited. The latter appears to drive indeterminate growth dynamics in *P. ochraceus*. Individual-based mechanistic models, which can illuminate underlying physiological responses, offer a viable framework for forecasting population dynamics in the keystone predator *Pisaster ochraceus*. The DEB model herein represents a critical step in that direction, especially in a period of increased anthropogenic pressure on natural systems and an observed recent decline in populations of this keystone species.

## Introduction

Improving our ability to anticipate responses of natural systems to environmental change is among the most pressing challenges facing modern ecological theory [1]. Efforts have been confounded by the inherently complex nonlinear dynamics of such systems [2]–[4]. However, the physiological responses of individuals may be considered as the underlying basis of all ecological dynamics, thus providing a solid foundation for advancing the field of ecological forecasting [1]. Studies at the organismal level have emphasized that some of the first responses to climate change may lie not in mortality but in changes in growth and reproduction [5], [6] and in the strength of species interactions [7]–[9]. Particularly promising are bioenergetics studies that quantify flows of energy and mass through an individual, which in turn dictate levels of physiological performance including feeding, growth and reproduction. This provides a mechanistic framework that can help characterize physiological responses to current and projected environmental drivers as a consequence, for example, of increasing temperatures [10].

Predictive frameworks based on bioenergetics have been used for a wide range of species from a variety of taxa, and range in complexity from fairly simple to very elaborate [11]. However, given the complex nature of some of the threats currently faced by natural systems (e.g. climate change, ocean acidification, pollution), where intertwined direct and indirect effects can impact multiple species simultaneously, the most efficient approach may be to concentrate on ecologically important players, whose dynamics can exert cascading effects on populations and communities [7], [12]. Following this reasoning, keystone species [13], [14] may serve as ideal candidates for investigating and modeling the physiological mechanisms that ultimately mediate ecological processes [15]. Particularly, keystone predators – consumers that can remove competitive dominants or otherwise have impacts on an ecosystem disproportionate to their abundance [14], [16], [17] – have received much attention. Despite our generally good understanding of the links between the physiological condition of many species and their interactions with their environment (i.e. eco-physiology), few quantitative physiological models have been developed for keystone predators, and specifically there is a pressing need for models of feeding, growth and reproduction, and their response to changes in environmental drivers [18].

Here we describe a Dynamic Energy Budget (DEB), an individual-based mechanistic energetics model [11], [19], for the quintessential keystone predator, the rocky-intertidal sea star *Pisaster ochraceus* (Brandt 1835) (hereafter, *Pisaster*). By preferentially foraging on a dominant space-competitor, the mussel *Mytilus californianus*, *Pisaster* has profound impacts on intertidal community assemblages [14], [20]. Exploiting the virtues of DEB theory, we describe a model that can (1) predict *Pisaster* growth at larval and post-metamorphic stages when prey are abundant and available *ad libitum*, (2) characterize shrinkage when food is removed, and (3) illuminate dynamics in physiological processes driven by cumulative effects of temperature and prey availability. This model represents a critical first step in exploring, and forecasting how variation in environmental drivers will likely affect the physiological performance and rates of foraging of this keystone predator [21]. Such an understanding is especially timely given the recent widespread mortality of *Pisaster* being observed on the Pacific coast of North America (Eric Sanford, pers. comm.).

While several bioenergetics models seeking to relate metabolic organization to aspects of physiological performance exist, DEB theory is gaining increased popularity because of its ability to model underlying physiological processes (maintenance, growth, development, and reproduction) based on first principles, that are common to all life forms including different taxa and life stages [22]. Unlike net-production models (e.g. scope for growth), which maintain that assimilated energy is partitioned between maintenance and growth/reproduction, DEB theory assumes that energy is first stored as reserves, and then distributed among physiological processes [23]. This topology offers solutions for multiple biological problems [11], three of which we emphasize here given their importance for *Pisaster*. Firstly, we rely on the capacity of the DEB to mechanistically describe the whole life cycle of a generalized organism without having to modify the structure of the model throughout ontogeny [24]. This is accomplished by explicitly accounting for energetic requirements associated with the life-history processes of maturation and maturity maintenance. Incorporating these costs is non-trivial from both physiological and ecological standpoints, as highlighted by a growing body of literature revealing that challenges faced by individuals early in life can impair performance at later stages [25]–[29]. Since the keystone role of *Pisaster* is restricted to its benthic life stages, efforts to model the influence of environmental variables on its physiological condition have mainly focused on post-metamorphic stages ([15], [30], [31] but see [32]). Notably, however, an important portion of its existence occurs as a planktotrophic larva [33]. The model presented here exploits the capacity of DEB theory to account for maturation and maturity maintenance and, building upon available data for both larval [32] and post-metamorphic stages [34], provides a means for simulating growth trajectories of *Pisaster* throughout ontogeny.

Secondly, a reserve compartment provides organisms with a physiological buffer against environmental fluctuations, by which vital rates and dynamics of structural mass are partially independent of changes in prey availability. DEB theory thus offers a framework for accounting for time history aspects of environmental signals. Weight-loss and shrinkage (i.e. reduction in structure to pay for somatic maintenance [11]) are common for some intertidal organisms such as annelids, echinoderms, and cnidarians [34]–[37] frequently having to cope with severe energy limitations due to abiotic (e.g. waves, heat and desiccation stress) and biotic conditions (e.g. competition, low prey availability). In an attempt to improve the accuracy of the model with respect to starvation, we include an additional parameter calibrated using data from controlled laboratory observations.

Thirdly, organisms rarely face single stressors in nature [38]; instead, the environment tends to challenge individuals through cumulative effects of multiple factors. As has been well established, the relative importance of predatory species on their communities is largely determined by their sensitivity to varying conditions of body temperature and food [39]–[43]. Surprisingly, despite widespread recognition of the critical ecological role of keystone predators, few models have been developed that account for the interactive effects of these variables on their physiological condition. Developing such models is particularly necessary for species experiencing extreme variability in environmental conditions. Throughout its wide range of distribution along the west coast of North America (between Alaska and Baja California), *Pisaster* encounters large temporal and spatial variation in temperature and prey availability, so a model capable of accounting for the cumulative effects of simultaneous changes in these variables should prove especially useful. If we are to predict responses of individuals to natural and/or anthropogenic pressures it is therefore crucial to account for multiple sources of stress [44]. Due to logistic and conceptual challenges, designing experiments that provide comprehensive, yet easy-to-interpret data has troubled eco-physiologists hoping to bridge the gaps between empirical observations and estimates of fitness [45]. Based on individual bioenergetics, DEB theory provides a general (i.e. non taxon-specific) framework that can be utilized to uncover physiological mechanisms by which multiple stressors combine to impact performance in organisms [11], [45], [46]. To incorporate these effects, the model described here is based on empirically-derived estimates of temperature sensitivity, feeding functional response, and starvation dynamics of *Pisaster*.

The DEB model builds on both observational studies, which provide information of the basic biology of *Pisaster*, and manipulative studies addressing the effects of changes in body temperature on metabolic, feeding, and growth rates. These data were obtained both from the literature and from our own experiments, which were especially designed for DEB modeling purposes. Our aim is to provide an individual-based mechanistic model that can characterize the physiological condition of *Pisaster* throughout ontogeny, and in response to cumulative effects of changes in body temperature and prey availability across its geographic range.

## Model Description

**Figure 1:**
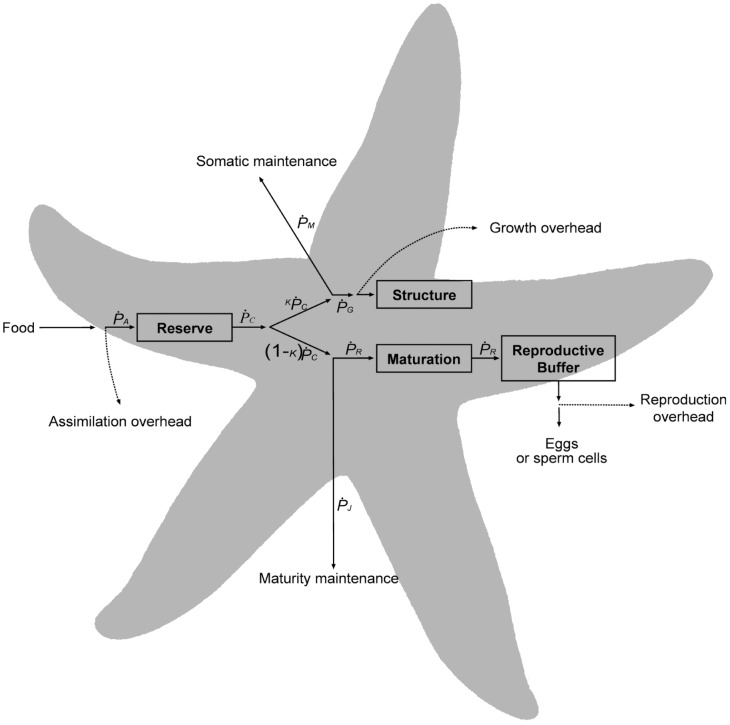
Schematic representation of standard Dynamic Energy Budget model. Arrows represent energy fluxes (J d^−1^) that drive the dynamics of the four state variables, depicted in boxes (Reserve, Structure, Maturation, and Reproductive Buffer). Energy enters the animal as food, and then assimilated at a rate 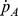 into Reserves. Mobilization rate, 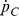, regulates energy fluxes to cover the demands from somatic maintenance, 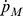, structural growth, 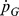, maturity maintenance, 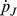, maturation, 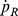 (immature individuals), and reproduction, 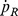 (mature individuals). The parameter kappa (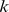) is the proportion of mobilized energy diverted to 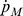 and 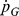, while the rest (1−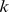) is used for 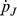 and 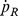. Formulations explaining these fluxes are given in the Appendix S1. Overheads associated to assimilation, growth and reproduction arise due to thermodynamic inefficiencies when transforming between substrates.

Dynamic Energy Budget (DEB) theory describes energy and mass flows in an individual organism (Fig. 1) throughout its life history. In its purest form DEB considers an archetypal individual that is representative of all individuals of the species, although several authors have extended the theory to examine intraspecific variability, such as occurs along latitudinal gradients [39]. The model herein was first developed following the assumptions of a standard DEB model (i.e. one reserve compartment, one structure compartment, isomorphic growth). While excellent comprehensive descriptions of the standard DEB model and its fundamentals are provided elsewhere [11], [22], [47], we offer a basic explanation of the formulations that orchestrate our generalized model in the Appendix (S1). As illustrated in Figure 1, the model tracks dynamics of four state variables (reserve, structure, maturation, and reproductive buffer), which depend on energy flows (units of J d^−1^; represented by arrows). Energy assimilated from food at rate 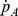, first enters the reserve compartment. Energy can then be mobilized at rate 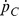, and allocated depending on the parameter kappa (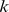) [11], [19], which amounts to a fixed fraction of energy used for somatic maintenance at rate 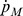, plus growth at rate 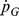. The remainder, 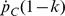, goes to maturity maintenance at rate 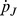, plus reproduction at rate 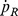.

The standard DEB model (Appendix S1) was modified to incorporate relevant aspects of *Pisaster* life-history. Specifically, we accounted for growth during larval stage, the ability of individuals to shrink (i.e. compensate for somatic maintenance costs using structure) when starved, and species-specific rules for energy expenditure in spawning. The steps taken to incorporate these aspects into the standard model (Appendix S1) are detailed below.

### 2.1. *Pisaster ochraceus* DEB model structure

Since relevant information for the different life-stages of *Pisaster* was available in the literature, it was possible to build a model that encompasses the whole life-span of a generalized individual, accounting for changes in morphology, energy allocation rules, and growth patterns that follow when transitioning between stages [48]–[50].

Including a larval stage implies deviations from the standard DEB model due to violations of the isomorphy assumption arising from the stark morphological differences between *Pisaster* larval and post-metamorphic stages (planktonic ciliated swimming larva vs. benthic juvenile and adult). Standard DEB models use one shape coefficient, 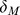, to convert physical lengths, 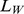 (e.g. larval length), to structural lengths, 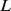 (a useful theoretical measure of size that directly relates to the state variable structure and is not influenced by the organism’s shape), through the equation 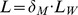. Because morphology differs between the larval and post-metamorphic stages, the relationship between physical and structural length needs to be described independently for each stage, which we do here by estimating two shape coefficients, 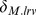 and 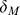, respectively. Violating the isomorphy assumption also implies that surface-area is proportional to volume^1^ instead of volume^2/3^– as for isomorphs [51]. As a consequence, growth during larval development is accelerated [32], which is therefore better described by an exponential rather than the asymptotic von Bertalanffy growth model [51]. Indeed, using data from George [32] and Pia et al. [52], we found that larval surface-area was proportional to volume^0.97^, an exponent that is not statistically different from 1.0. It has been argued that, as a result, the processes of assimilation and mobilization rates (Appendix S1, equations 1 and 3, respectively) increase during larval development [11], [48]. Since somatic maintenance is proportional to volume (Appendix S1, Eq. 4), there is no limit to the increase in structure [51], in agreement with observations [32], [48], [49].

The increase in both processes 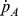 and 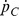 during the larval phase has been modeled by means of a shape correction function, 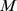
(following [48]):
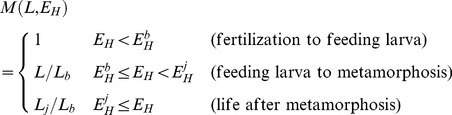
(1)where 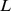 is structural length (cm) and 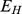 is energy allocated to maturation (J). 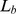 and 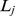 correspond to structural lengths (cm) at birth and metamorphosis, respectively. Parameters 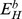 and 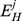 are defined as the energy invested in maturity (J) for reaching “birth” as a feeding larvae and metamorphosis, respectively (Table 1). Because 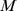 is applied to those processes containing the parameters 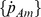 and 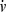 (Appendix S1, equations 1 and 3), it may strongly influence all processes that depend on them. Importantly, it will have an impact on the expected asymptotic body length, 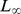 [51].

**Table 1:** *Pisaster ochraceus* DEB parameter values, and results of sensitivity analysis.

Parameter	Symbol	Value±SD	Units	Sensitivity
**Primary parameters**				
Half-saturation coefficient^1^	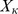	13.9±2.3	mussels m^−2^	**−0.01**
Maximum surface area-specificassimilation rate^2^	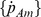	43.2±4.1	J d^−1^ cm^−2^	0.20
Energy conductance^2^	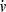	0.04±0.01	cm d^−1^	0.07
Fraction of energy used for somaticmaintenance and growth^2^	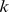	0.58±0.07	–	0.11
Volume-specific costof maintenance^2^	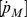	40.43±1.41	J d^−1^ cm^−3^	**−0.14**
Volume-specific costof maintenanceduring starvation^1^	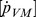	11.5±2.74	J d^−1^ cm^−3^	0.00
Volume-specific cost of structure^2^	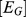	2743±97.22	J cm^−3^	0.00
Maturity at birth^2^	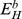	0.012±4.8×10^−4^	J	**−0.03**
Maturity at larval settlement^2^	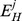	100±4.21	J	0.00
Maturity at puberty^2^	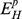	13.9×10^6^±99×10^6^	J	0.00
Shape coefficient of larvae^2^	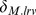	0.959±144.56	–	0.00
Post-metamorphic shapecoefficient^1^	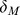	0.52±0.03	–	**−0.09**
Maturity-maintenance ratecoefficient^2^	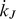	2.9×10^−6^±0.018	d^−1^	0.00
**Temperature dependence**				
Arrhenius temperature^1^	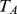	6000±335	K	**−0.02**
Lower limit of tolerancerange^3^	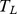	280	K	**−0.99**
Upper limit of tolerancerange^3^	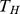	297	K	0.00
Arrhenius temperature atlower limit^3^	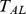	31000	K	0.01
Arrhenius temperature atupper limit^3^	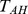	190000	K	0.00
Reference temperature^4^	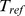	293	K	NaN
**Conversion parameters**				
Density of structure^4^	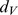	1	g cm^−3^	NaN
Weight-energy couplerfor reserves^4^	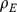	4.35×10^−5^	g J^−1^	NaN
Molecular weight ofreserves^4^	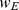	23.9	g mol^−1^	NaN
Chemical potential ofreserves^4^	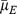	550	kJ mol^−1^	NaN

1 Estimated directly from data.

2 Estimated using covariation method (DEBtool).

3 Estimated using grid-search.

4 Kept fixed.

Sensitivity is the percent change in arm length at age 2 y divided by the percent change in a single parameter value (10%). Analyses were carried out using *ad libitum* food, at a temperature of 13°C. Parameters with a negative relation to growth are printed in bold type. Sensitivity of parameters not estimated is NaN.

As is the case for many marine invertebrates (e.g. anemones, urchins), sea stars have indeterminate growth, and size dynamics may vary dramatically according to habitat conditions. When starved during extended periods these organisms lose weight [34], [35]. Initially, there is a reduction of stored reserves [53], [54], but once these are depleted, the overarching priority given to the process of somatic maintenance, 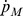, would presumably lead to a reversing of energy/mass flux from structure to cover the costs of living, and the organism shrinks (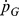 becomes negative, Fig. 1) [11]. The assumption that somatic maintenance is prioritized has been empirically confirmed for *Pisaster ochraceus* [55], [56] and its congener, the subtidal *Pisaster giganteus* [57]. Histological studies with *Pisaster* further revealed that during prolonged starvation energy reserves contained in the pyloric caecum decrease to levels insufficient for gonad production [55], [56], thus compromising reproduction in favor of somatic maintenance.

Due to thermodynamic constraints, mobilizing energy from structure to somatic maintenance is less efficient than mobilizing it from the reserve compartment [11], [22]. To account for the physiological adjustments during periods of prolonged starvation (i.e. when mobilized energy cannot cover somatic maintenance, 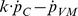), we introduced a new parameter, 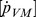
(J d^−1 ^cm^−3^), which adjusts the rates at which structure shrinks, -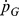, and somatic maintenance is paid, 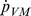
(J d^−1^):
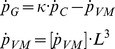
(2)


Also, to characterize the effect of starvation on maturity and maturity maintenance, we followed the approach used by Augustine et al. 2011 [58]. During periods when mobilized energy cannot cover maturity maintenance, i.e. 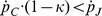, change in maturity (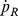; Appendix S1, Eq. 8) is calculated as:
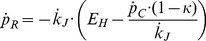
(3)


The rules for emptying the reproductive buffer are defined based on species-specific considerations. Evidence shows that gametogenesis in *Pisaster* is driven by annual changes in photoperiod [59]. Gonadal volume increases towards the winter months, and gametes are released during late spring and early summer depending on latitude [60]–[62]. Our model makes the simple assumption that all individuals empty their reproductive buffer as gonads every 365 d.

### 2.2. Going from the DEB model to traditional metrics of growth and reproduction

DEB model quantities can be converted from more traditional metrics reported in the literature to estimate parameter values used in the model. Conversely, comparison of metrics generated from DEB to traditional metrics (not used in model parameterization) provides an opportunity to independently train and validate model outputs. Two commonly used metrics of the size of sea stars are arm length, 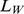 (cm), and wet weight, 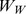 (g). Arm length can be obtained from the quotient between structural length and shape coefficient (Appendix S1). Wet weight is calculated from structure, reserve and reproductive buffer [11]:
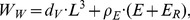
(4)where 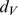 (g cm^−3^) is density of structure, assumed to equal 1, and 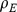 (4.35·10^−5^ g J^−1^) is weight-energy ratio for a generalized reserve molecule [63], calculated from the per carbon atom molecular weight 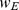 (23.9 g mol^−1^) and chemical potential of reserves 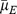 (550 kJ mol^−1^): 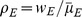. Note that 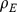 transforms energy to weight of reproductive buffer as well.

Additionally, estimates of reproductive potential are often employed as proxies for fitness. Reproductive potential in asteroids, commonly known as Reproductive Output index (*RO*, dimensionless) or Gonadal Index, the ratio between the gonadal and somatic mass [61], [62], [64], can be described in DEB terms by the following equation:
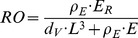
(5)


## Parameter Estimation and Model Training

The DEB parameter values for *Pisaster* were estimated by the covariation method [63], [65] implemented in the MATLAB 2010 software package DEBtool (available at http://www.bio.vu.nl/thb/deb/deblab/debtool/), which employs a Nelder-Mead numerical optimization to minimize the difference between observed and predicted values based on a weighted least-squares criterion. The estimation procedure simultaneously uses both real data from observational and manipulative studies and pseudo-data from theory in the parameter fitting process [48], [66]. This approach is possible because DEB theory is formulated under the premise that all living organisms regulate metabolic processes using more or less the same mechanisms. Given this assumption we can describe these processes with a set of DEB parameters, and it follows that differences between species are underpinned by variations in parameter values among common mechanisms [63].

The covariation method can accommodate diverse data sets that provide information about the basic biology of the target species, including size/age at transitions between life stages, growth, feeding, or reproductive output measurements, as well as data sets generated to estimate DEB theory quantities. We used the covariation method to (1) estimate DEB parameters for which we had no real data (e.g. 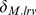), and to (2) optimize the estimates obtained for parameters we determined empirically (e.g. 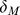) (Table 1). Our training phase used field and laboratory measurements of size at age, laboratory functional response data, field and laboratory measurements of reproductive output, and laboratory measurements of thermal sensitivity of metabolism. The data sets used for parameterizing and training the DEB model for *Pisaster* are detailed below. All information collected from figures found in the literature for which no data tables were provided was extracted using *DataThief III* [67]. All animals used for experimental and observational purposes were collected with permission granted by the California Natural Resource Agency, Department of Fish and Game (Scientific Collection Permit, ID Number: SC-11078).

### 3.1. Data sets

#### 3.1.1. Growth and shrinkage

Growth time-series are of great value for estimating DEB parameters, but only if accurate body temperature and food availability data are also available [10], [68]. Because body temperature and food availability data are often limited, parameter estimations may be based on observations made over short time windows. This reduces confidence in the model’s ability to simulate performance over prolonged periods of time, where digestion limitations are possibly defining maximum feeding and growth rates [69]. We used growth data for the larval and adult stages available from George [32] and Sanford [21], respectively. Data retrieved from both sources were collected from individuals fed *ad libitum* (i.e. *f* = 1), and both studies reported water temperatures. Changes in larva width, 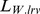
(cm), were used as a metric of larval growth, while changes in arm length, 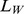 (cm), were used to assess growth during post-metamorphic stages.

We conducted a laboratory experiment to quantify long-term changes in size during starvation (i.e. *f* = 0), and ultimately to determine the parameter 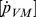. Mature individuals (∼100 g) were kept in a 2600-L recirculating seawater tank (temperature controlled at 12°C; provided with a protein-skimmer; water chemistry monitored every other week and partial water changes conducted accordingly) for 467 d (N = 5) and 152 d (N = 1), and wet body weight, 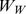
(g), was measured at irregular intervals ranging from 1 to 10 wk. Data collected for each individual were compared to DEB predictions obtained from the parameterized model. Values of 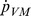 were adjusted until a minimum deviation between observations and predictions was found, based on a root-mean-square error (RMSE) criterion. Shrinkage volume-specific cost of maintenance during prolonged starvation, 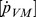, values from all individuals were averaged to determine the overall best estimate.

#### 3.1.2. Life-stage transitions

Growth data were complemented with information about size and age at transitions between stages: “birth”, defined as the onset of larval feeding, occurs around day 9–10 after fertilization [60], when 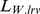 = ∼0.03 cm (12°C) [32]; larvae reach competency to metamorphose and settle after ∼50 d post-fertilization (12–15°C) [70]; and puberty has been estimated under field conditions around age 5 y, when wet weight is ∼70–90 g [71].

#### 3.1.3. Reproductive potential

Reproductive potential can be estimated from studies conducted in the field or in the laboratory, as long as relative levels of resource availability are known (e.g. [48], [72]). We used field data from Sanford and Menge [61]; specifically the highest value for Reproductive Output index reported, i.e. *RO* = 0.23. Similar values have been reported from laboratory experiments where *Pisaster* was given *ad libitum* food supply [59].

#### 3.1.4. Feeding functional response

We estimated the half-saturation coefficient 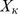 through a mesocosm experiment conducted at Bodega Marine Laboratory (BML, UC-Davis) in July 2012. Feeding rates of individual sea stars (200 g wet weight) were measured in five food density treatments (5, 11, 21, 32, and 48 mussels m^−2^; *Mytilus californianus*; 2-cm shell length). Five 300-L tanks supplied with running seawater were each divided in fourths (0.57 m^2^) to allow for 20 simultaneous feeding rate observations. Sea stars were collected at Bodega Bay, CA (38°18′16″ N, 123°03′15″ W) and kept individually under running seawater for one week prior to the experiment. Individuals were starved for six days, and fed *ad libitum* on day seven to standardize hunger. On day eight each animal received a randomly chosen food density treatment, and was allowed to forage for seven hours. Eaten mussels were then quantified and their tissue dry weight determined from an empirical relationship based on mussel shell length: 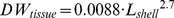 (N = 98, r^2^ = 0.98). Feeding rates, expressed as consumed 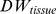 h^−1^, were then scaled by the maximum value to obtain *f*. The relationship between food density and *f* (Appendix S1, Eq. 1) was fitted using a non-linear least-square regression, which yielded an estimate for 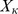.

#### 3.1.5. Temperature sensitivity

The sensitivity of *Pisaster* to changes in temperature was determined from O_2_ consumption measurements taken in five water temperature treatments: 10, 14, 18, 20, 24 and 26°C. Sea stars (mean ± SE = 105.4±5.2 g wet weight, N = 48) were collected at Bodega Bay, CA (38°18′16″N 123°03′15″W) and kept in tanks with running seawater (10.8±0.7°C, mean ± SD) and *ad libitum* food supply (*Mytilus californianus* mussels) at BML for 5 d before experimental temperatures were adjusted. Pairs of individuals were then transferred to 60-L aquaria filled with 1-µm filtered seawater at ambient temperature (∼12°C). Experimental water temperatures were achieved by keeping the aquaria in climate-controlled rooms. The two highest treatment temperatures were reached using 100-W aquarium heaters (Marineland Visi-Therm, USA). Water temperatures were changed at a rate of ∼1°C h^−1^. Individuals were kept at desired temperature treatments for 4 d before measuring O_2_ consumption rates. To maintain water quality, tanks were equipped with air-stones and submersible pumps. Water chemistry (salinity, pH, ammonia, nitrite, and nitrate) was monitored every other day using a saltwater test kit (API, USA), and partial water changes were performed when needed (every 1–2 d). Individuals were then placed in cylindrical watertight chambers (2.88 L) filled with aerated, 1-µm filtered seawater, at its corresponding treatment temperature. A magnetic stir-bar kept the water circulating during measurements. A Clark-type electrode (HANNA-9143, USA), fitted over the top of each chamber, was used to measure dissolved O_2_ concentration (ppm) at 10 and 40 min after sealing the chamber. Trials were terminated early if oxygen concentration dropped below 70% of the initial reading. The change in O_2_ content was standardized by the animal’s dry mass. For each temperature treatment, two sea star-free chambers were used as blanks to account for background changes in O_2_ concentration.

The temperature sensitivity experiment was run twice (August 2011 and July 2012). This data set was complemented by measurements of growth rate taken at ∼5°C by Gooding et al. [31]. These data were then used to optimize thermal sensitivity parameters (Table 1). Arrhenius temperature, 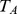, was estimated from the slope of an Arrhenius relationship [39] using measurements taken at 10, 14, 18 and 20°C. Once 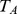 was known, a grid-search was conducted to find the combination of parameter values for 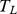, 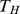, 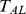, and 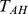 that minimized the RMSE between observed and simulated data. Maximum and minimum parameter values evaluated by the grid-search were determined by the range of values reported for a collection of species modeled through DEB, available on-line (http://www.bio.vu.nl/thb/deb/deblab/). The fitted curve was then scaled in relation to its maximum value to force the curve’s maximum through one.

#### 3.1.6. Post-metamorphic shape coefficient

The post-metamorphic shape coefficient, 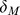, of *Pisaster* was first estimated from the empirical relationship: 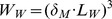, described using data of arm length (cm) and wet weight (g) from 457 individuals collected at Bodega Bay (38°18′16″ N, 123°03′15″ W). The estimate obtained from this analysis was then treated as an initial value in the covariation method. The new optimized estimate provided a closer approximation of the contribution of structure to body weight.

#### 3.1.7. Parameter sensitivity analysis

A parameter sensitivity analysis was carried out by varying each parameter by 10% and quantifying the percent effect on observed length at age 2 y. Sensitivity is the ratio of the percent change in length at age 2 y to the percent change in the parameter. This is equivalent to the partial derivative of length with respect to variation in a single parameter.

## Model Validation

Having estimated model parameter values for *Pisaster*, we validated the model predictions against growth data from 24 adult and juvenile sea stars kept individually by Feder [34]. His data were chosen because they are the only long-term time series available (∼1.6 y), produced using individuals kept under controlled laboratory conditions; food was provided *ad libitum* and water temperature is reported. Additionally, since growth was measured as a change in length and weight, we could use these data to evaluate our model’s capacity to predict variation in body mass due to spawning events.

Because the estimated parameters varied around a mean (Table 1), we simulated 1000 possible growth trajectories resulting from combinations of parameter values sampled from normal distributions defined by their average and standard deviation (Table 1).

Statistical comparisons between observed and predicted data were performed using standard model skill metrics Mean Absolute Error (MAE), Mean Absolute Percent Error (MAPE), and Root Mean Square Error (RMSE), a conservative measure of the absolute magnitude of error [73]. Generally, we regarded a fit to be good when MAPE did not exceed 10%.

The statistical language R [74] was used to carry out all calculations.

## Model Results

### 5.1. Model training results

DEB model parameter values for *Pisaster* were successfully estimated through the covariation method using data from both, experiments conducted specifically to determine DEB quantities and from the literature (Table 1). Note that while some parameters could be estimated with high accuracy, others suffer from important variance. Given the generality of a model designed to characterize a broad range of physiological processes regulating life-history traits throughout ontogeny, it is expected that some parameters are harder to determine. In particular, maturity at puberty, 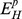, shape coefficient of larvae, 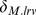, and maturity-maintenance rate coefficient, 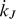, showed high variability (Table 1) because we lacked direct observations to estimate them. Future applications of this model should consider the uncertainties of these parameters, and possibly work towards reducing them.

**Figure 2:**
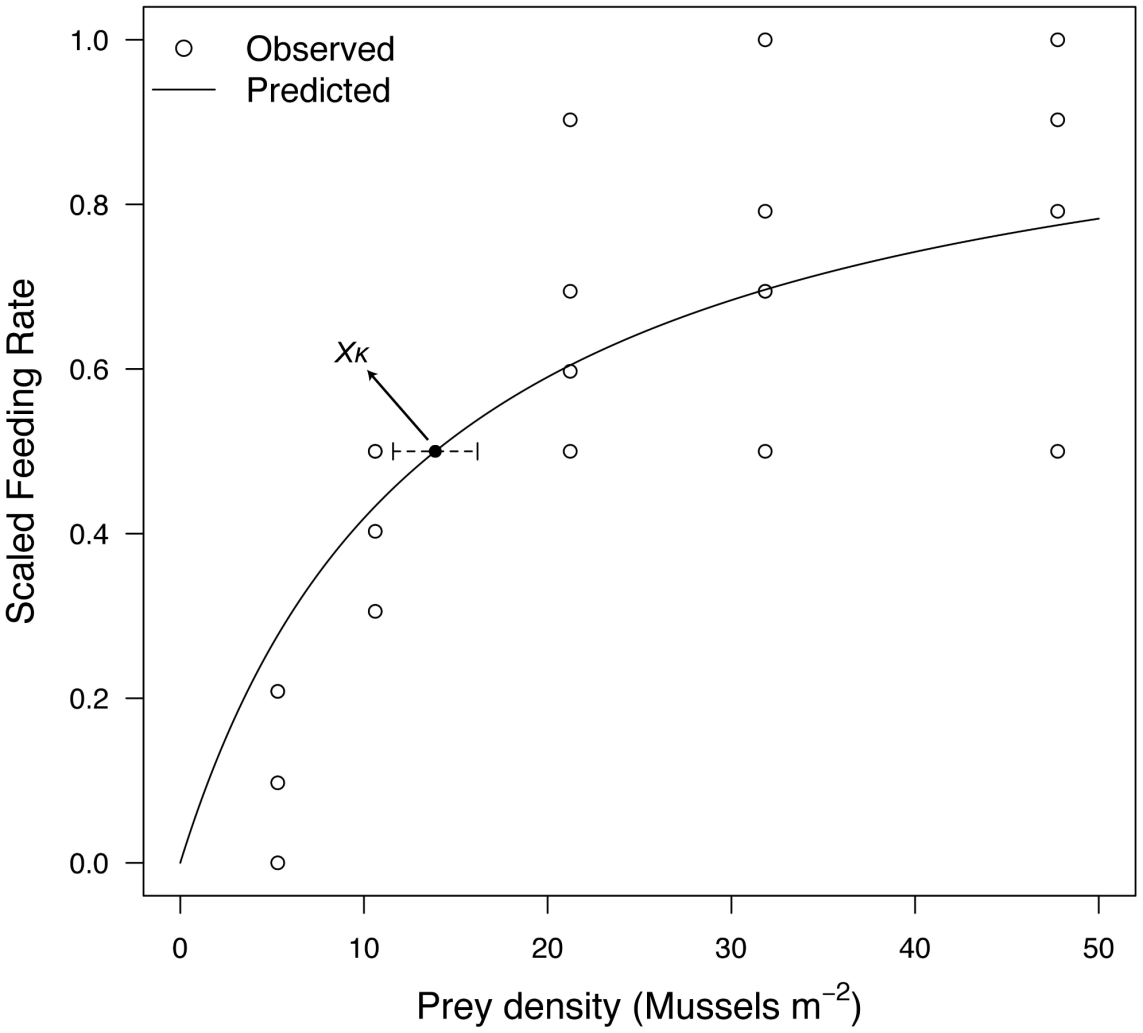
Scaled feeding rate as a function of prey density. Observed values (circles) and projection (line), based on a type II feeding functional response (Appendix S1, Eq. 1), are shown for mussels with 2-cm shell length. The estimated value for the half-saturation parameter 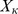 was 13.9±2.3 (Mean±1 SD) mussels m^−2^.

**Figure 3:**
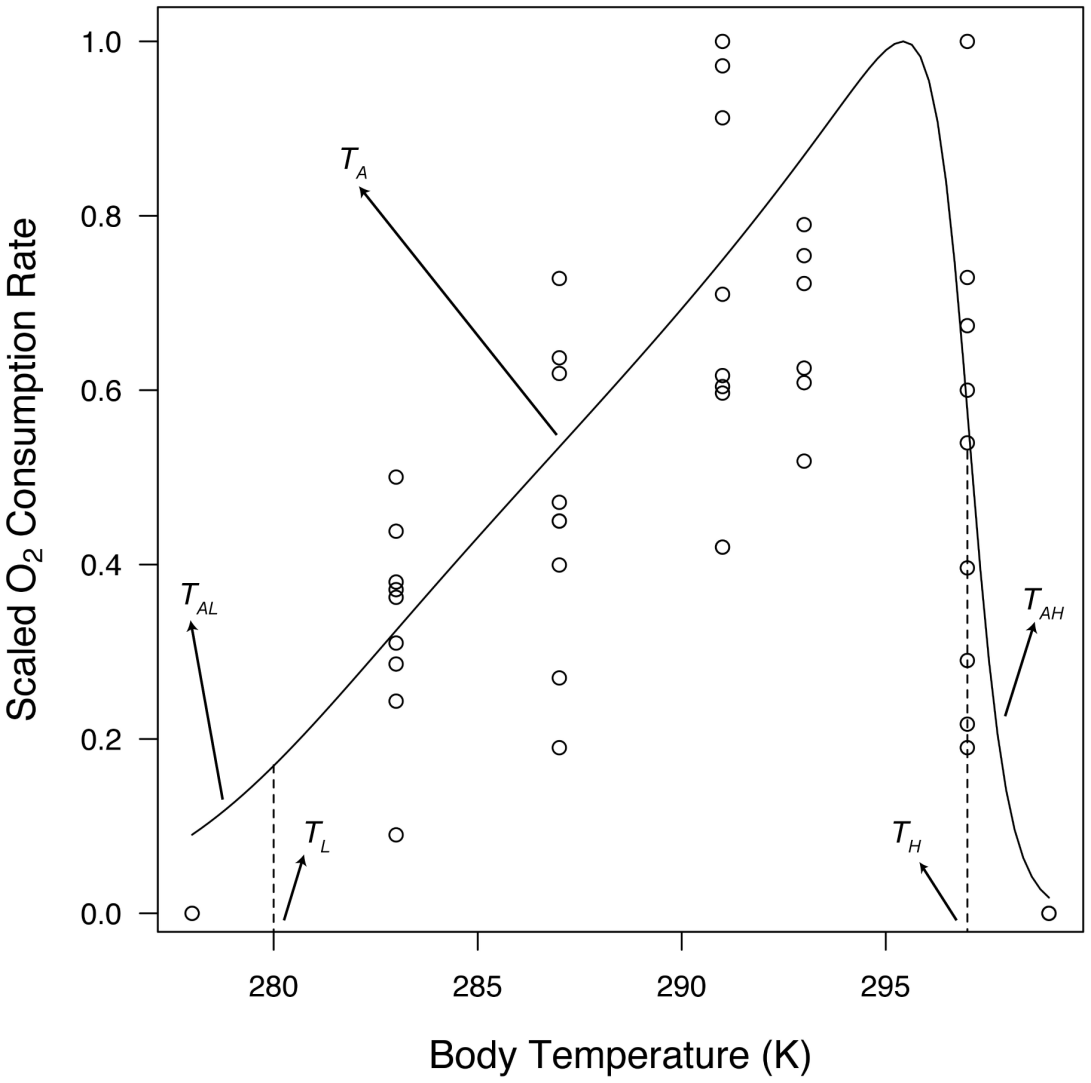
Temperature sensitivity. Observed values (circles) represent relative values of oxygen consumption and feeding rate (coldest temperature treatment) determined at a range of water temperatures from 278 to 299 K. The line of best fit was obtained by first estimating Arrhenius temperature, 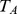, and then running a grid-search to find the combination of parameter values for 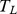 (lower limit of tolerance range), 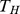 (higher limit of tolerance range), 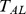 (Arrhenius temperature at lower limit), and 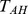 (Arrhenius temperature at higher limit) that minimized the RMSE between observed and simulated data.

**Figure 4:**
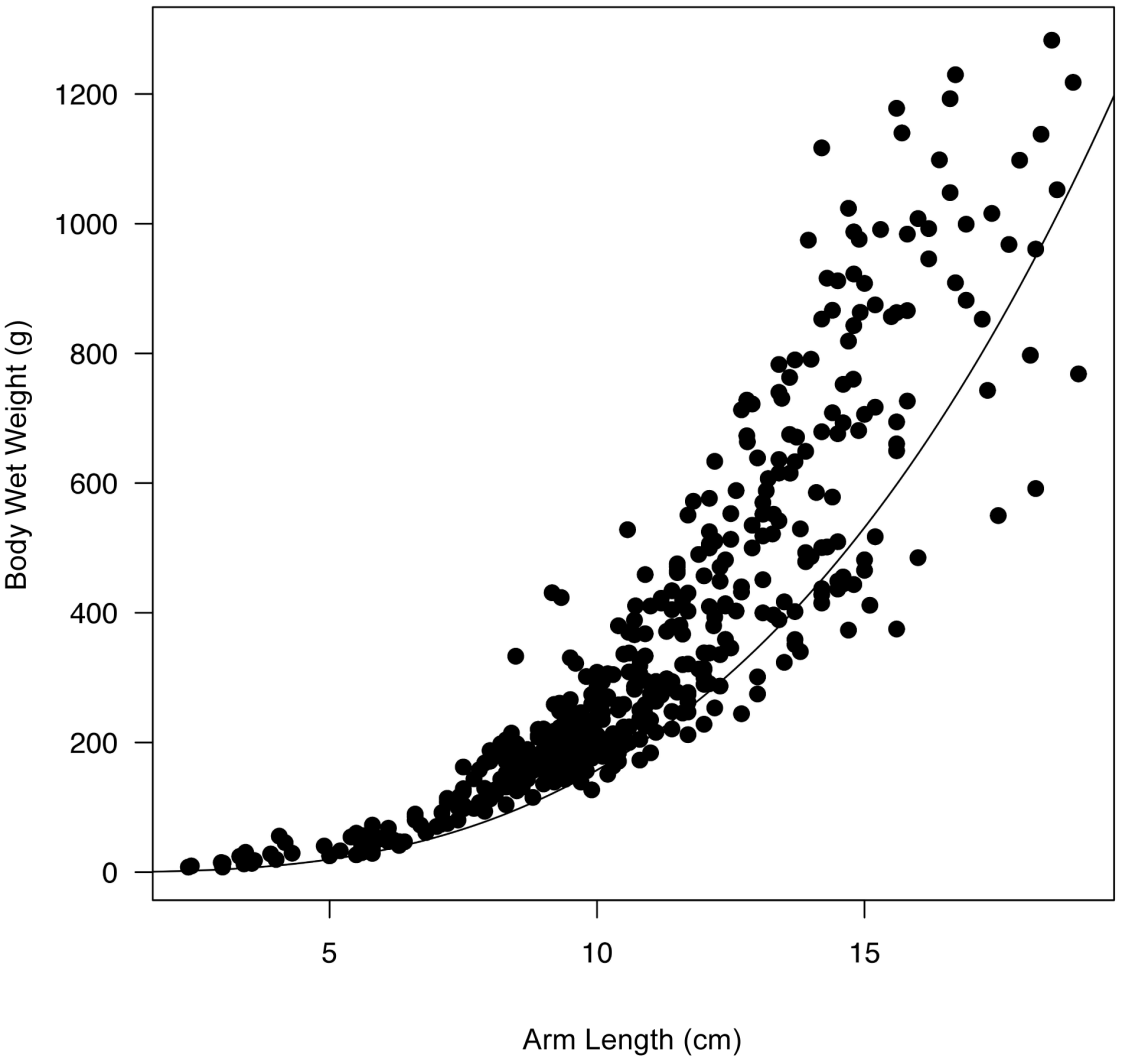
Body wet weight in (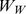) relation to arm length (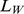). Observed values are shown as dots (N = 457 individuals). By fitting the equation 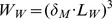, we estimated the post-metamorphic shape coefficient (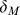). The estimate was then optimized through the covariation method (DEBtool), yielding 0.52±0.03 (Mean±1 SD). The trajectory described by this model is shown as a line crossing the cloud of points below their center, thus better representing the contribution of structure to body weight.

The half-saturation coefficient (section 3.1.4.), Arrhenius temperature (section 3.1.5.), and post-metamorphic shape coefficient (section 3.1.6.) were estimated directly from our data [68]. The non-linear least square regression from the feeding experiment yielded an estimate of 13.9±2.3 mussels m^−2^ for the half-saturation coefficient (Fig. 2). The grid-search for the thermal-sensitivity parameter yielded a RMSE between scaled data and model predictions of 0.22 (Fig. 3). The post-metamorphic shape coefficient, 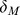, first empirically estimated to be 0.59±0.05, was then optimized with the covariation method, yielding a final value of 0.52±0.03 (Fig. 4).

**Figure 5:**
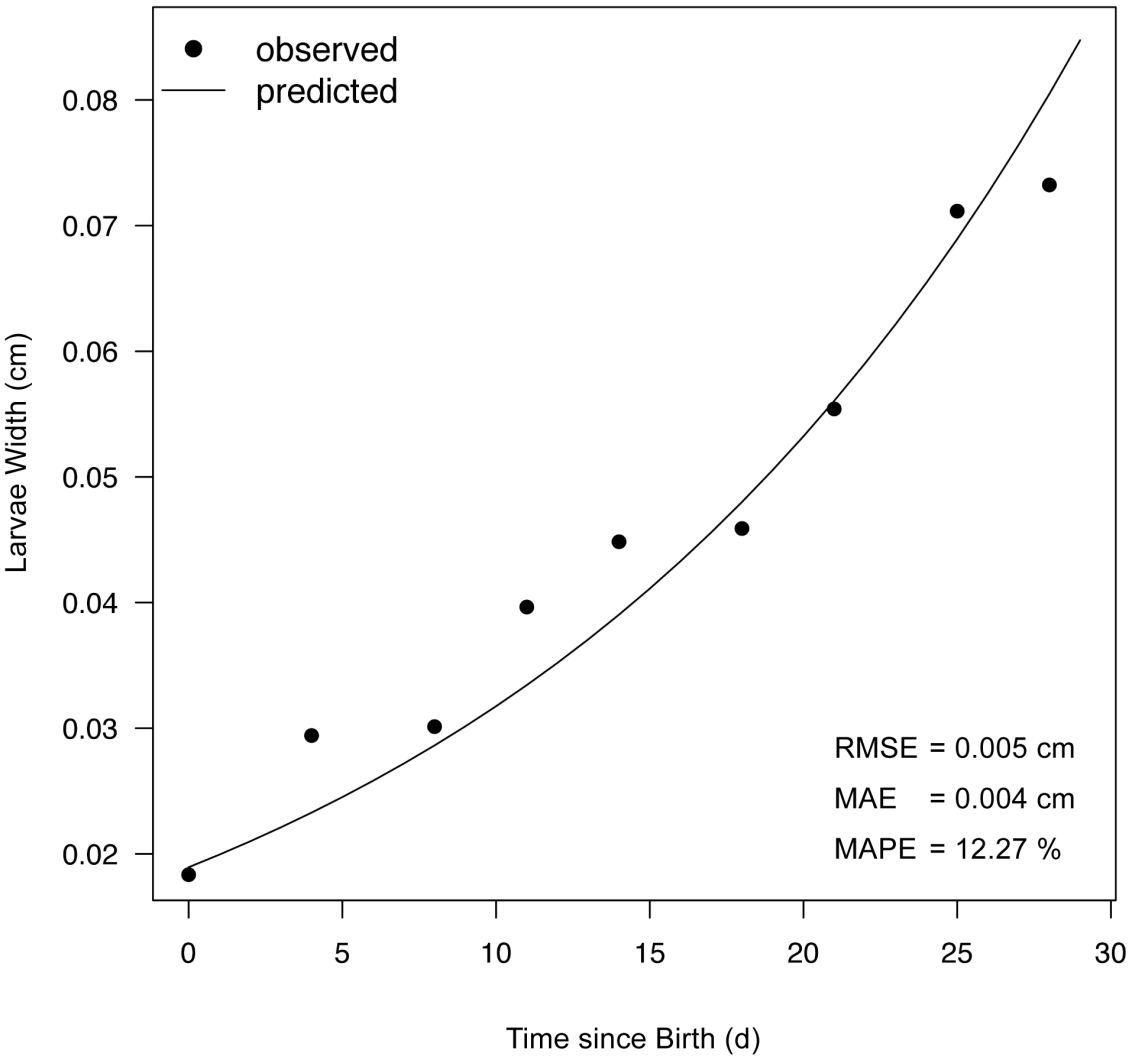
Larval growth from 0 to 27 d after birth. Birth is considered as the day when larvae begin feeding. Laboratory data (from citation [29]) are shown as dots. The line comes from a Dynamic Energy Budget model simulation, assuming *ad libitum* food and 12°C water temperature. Root Mean Square (RMS) error, Mean Absolute Error (MAE), and Mean Absolute Percent Error (MAPE) are shown.

**Figure 6:**
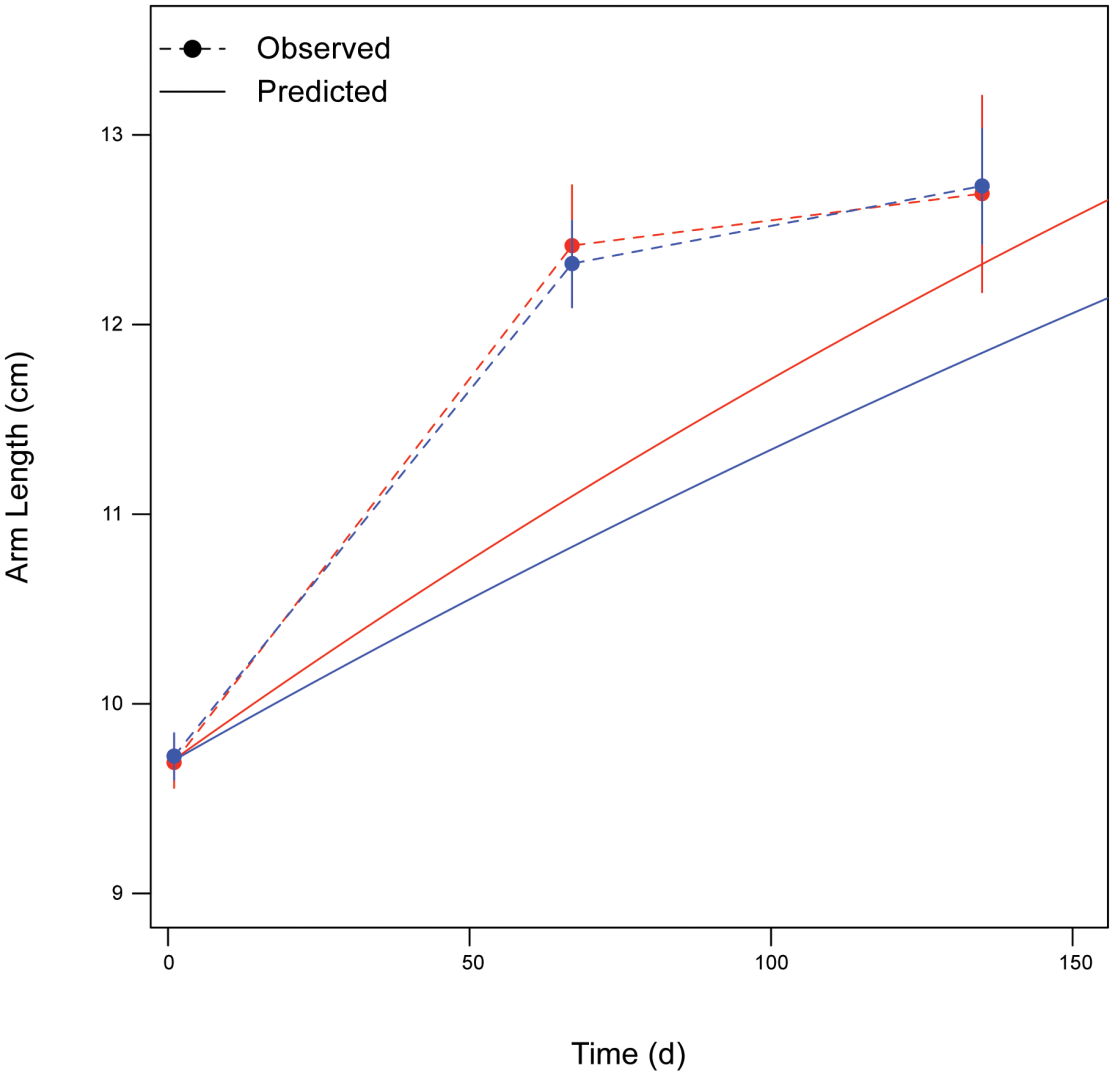
Post-metamorphic change in arm length over time at two water temperature treatments. Laboratory data from *ad libitum* feeding experiment (from citation [12]) are shown as dots. Solid symbols and black line are from 9°C treatment, open symbols and grey line are from 12°C treatment. Dotted lines are DEB predictions, grey levels as above.

We combined these empirically determined parameters with data from the literature**,** in an effort to simultaneously determine the remaining DEB parameter values using the covariation method (except for 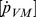, which was determined last) (Table 1), along with calibrating the model so it could capture important landmarks of the life-history of *Pisaster*, including size and age at transitions between life stages (sections 3.1.1. and 3.1.2.) and maximum reproductive output index (*RO*) (section 3.1.3.). Simulating ideal conditions (*f* = 1), the model predicted “birth” (first feeding larval stage) at 4.2 d after fertilization, when the larval size is 0.02 cm wide (vs. training values 9 d and 0.03 cm, respectively); settlement around day 59.9, when larval width is ∼0.38 cm (vs. training values 50 d and 0.37 cm, respectively); and puberty around day 264, when wet weight is ∼66.7 g (vs. training values 5 y and 70–90 g, respectively). The same simulation projects an estimate for *RO* of 0.21 (vs. training value 0.23). These predictions, along with the maximum size reported for *Pisaster* (20-cm arm length; [34]) allowed estimation of growth curves for both larval and post-metamorphic stages. The model’s ability to precisely track changes in larval body size (MAPE = 12.27%, RMSE = 0.005 cm) is illustrated in Figure 5. The comparison between observed and predicted growth data for the adult life stage further revealed the model’s good performance (overall RMSE = 1.01 cm) (Fig. 6). The training data for this adult stage were collected at two temperatures: 9 and 12°C [21] (section 3.1.1.). When running our model at each of these temperatures, agreement between observations and predictions was slightly better at 12°C (RMSE = 0.82 cm) than 9°C (RMSE = 1.18 cm). Although Sanford [21] did not find differences in growth between individuals kept at 9 and 12°C, our model’s built-in thermal sensitivity (independently estimated) predicts the 3°C difference in temperature would cause a significant change in growth (from 27 to 42% of maximal value). The lack of coherence between these model predictions, which suggest large changes on growth between temperatures on the steep part of the thermal performance curve, and Sanford’s data, which showed no difference in growth between 9° and 12°C, remains unexplained.

**Figure 7:**
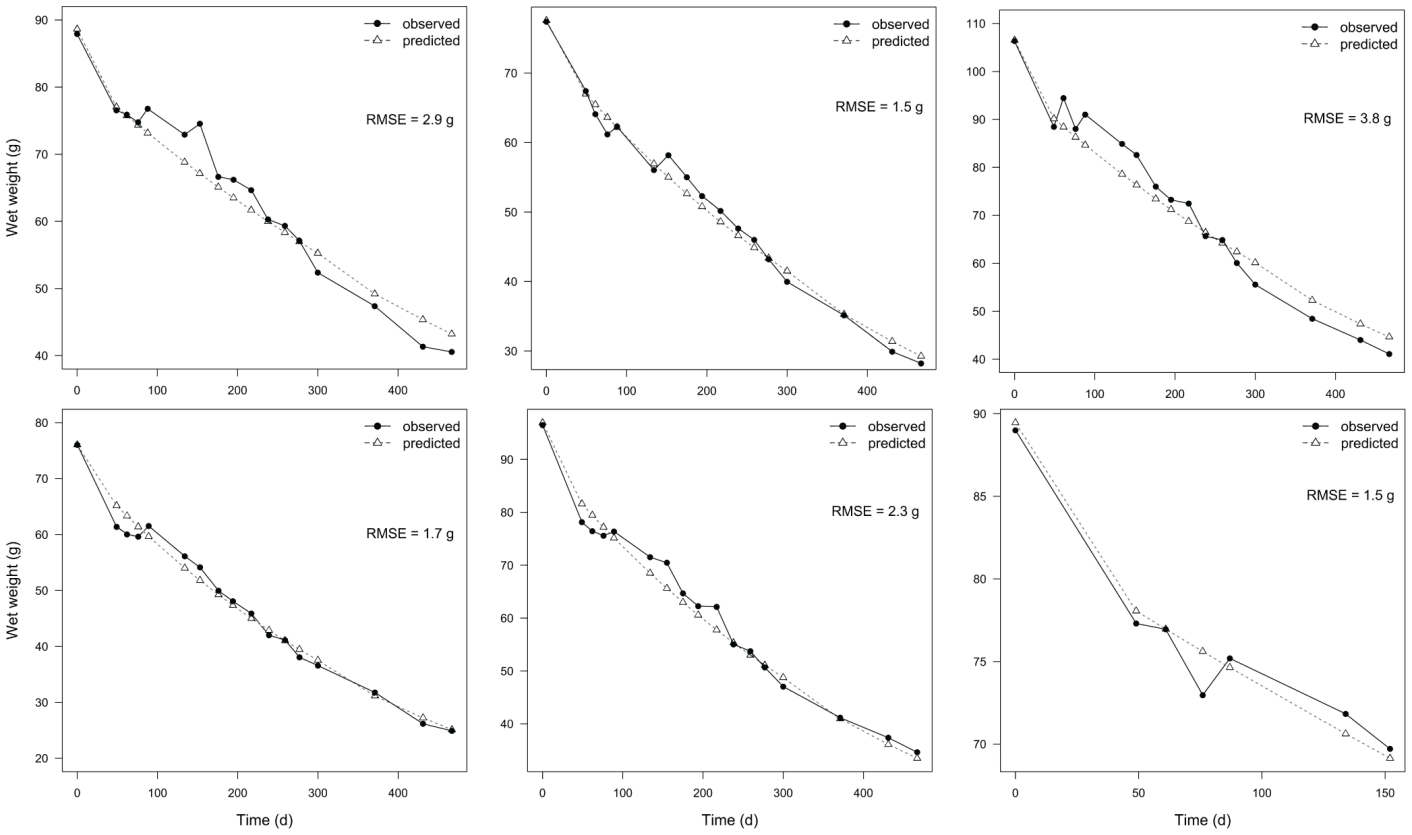
Post-metamorphic change in wet weight over time as a result of complete starvation. Each panel shows data for a different individual. Laboratory observations from long-term starvation trials are shown by dots and solid lines. Triangles and dotted lines are DEB predictions using the value for parameter 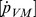 that minimized the RMSE between observed and predicted data. The mean of the six estimates of 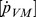, 11.5 J d^−1^ cm^−3^, was used in the DEB model.

Finally, our long-term starvation experiment together with the parameterized DEB model allowed estimation of the shrinkage volume-specific cost of maintenance parameter that applies during prolonged starvation, 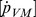 (Table 1). Individuals subjected to food deprivation lost weight at a steady rate of 0.12±0.02 g d^−1^ (mean±1 SD, N = 6). The values for 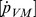 that minimized the RMSE between observed and predicted wet weight varied between 8 and 15 J d^−1^ cm^−3^ (Fig. 7). We used the mean, 11.5 J d^−1^ cm^−3^, as the value for this parameter.

### 5.2. Model validation results

**Figure 8:**
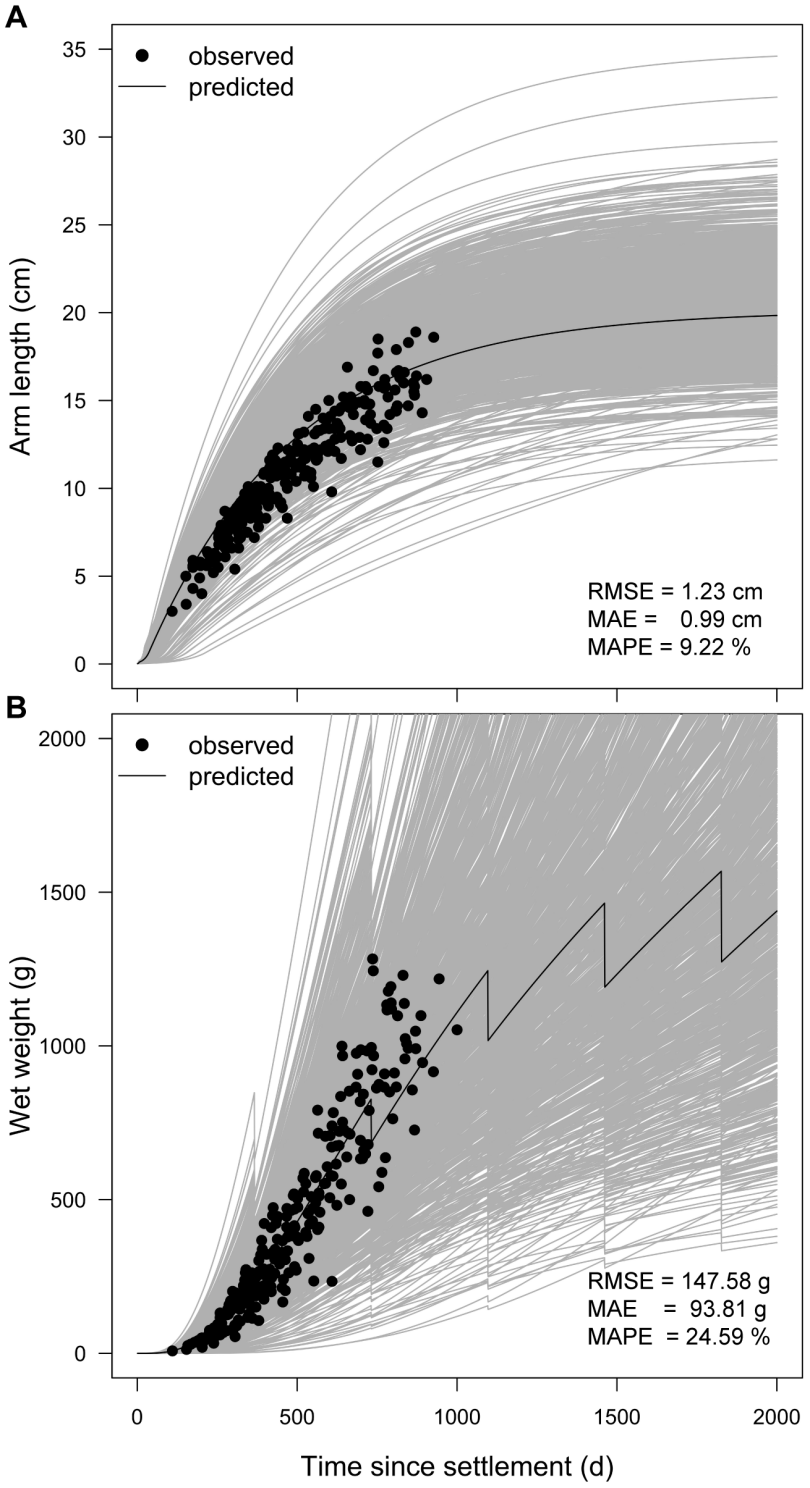
Post-metamorphic change in arm length and wet weight over time since larval settlement. Panel A illustrates arm length, and B wet weight. Laboratory observations (from citation [31]) are shown as dots. Food was provided *ad libitum*, and water temperature kept at 14.5°C, in accordance to the average reported by [31]. Grey lines are results of 1000 Monte Carlo DEB simulations, which simultaneously sampled parameter values from normal distributions with parameter means and standard deviations (Table 1). Black line is DEB simulation using mean values for all parameters (Table 1). Root Mean Square Error (RMSE), Mean Absolute Error (MAE), and Mean Absolute Percent Error (MAPE) are relative to the DEB simulation that used mean parameter values.

We ran the parameterized DEB model simulating conditions of food and water temperature, and compared the outputs to Feder’s [34] observations (Fig. 8). Similar to the conclusion obtained from the training protocol, the validation confirmed the model’s capacity to describe the increase in arm length of *Pisaster* through time, with an overall relative error MAPE = 9.22% (RMSE = 1.23 cm, MAE = 0.99 cm) when comparing observed data with the simulated growth trajectory obtained using the average parameter values (Fig. 8A). Note that agreement between observed and simulated data decreased with the size of the organism. The observed data lie within the envelope of the family of curves from the Monte Carlo simulations accounting for variability in parameter values and the simulations clearly track the change in arm length of *Pisaster* (Fig. 8A).

The model’s overall capacity to describe changes in wet weight appeared less satisfactory than for arm length (Fig. 8B). The indicator of relative error, MAPE, reaches 24.59% (RMSE = 147.56g, MAE = 93.81g) when comparing observed data with the simulated growth trajectory obtained using the average parameter values (Fig. 8B). The model’s lack of skill in predicting wet weight in *Pisaster* is further evidenced by the large spread of the family of growth curves from the Monte Carlo simulations that accounted for the variability in parameter estimates (Fig. 8B).

We performed a sensitivity analysis to evaluate the relative influence of the DEB parameters on *Pisaster* size at age 2 years (Table 1). Generally, the effect of increasing parameter values on the model output was approximately mirrored by the effect of decreasing the parameter values, and vice versa, indicating that most parameters had linear effects on growth. Effects were only nonlinear for thermal sensitivity parameters 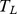 and 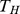. An increase in the value of the former had a strong negative effect on the model output (sensitivity −0.99), while a reduction caused a weak positive effect (sensitivity 0.04). In contrast, while increasing the value of the latter did not affect the model output, reducing it produced a strong negative effect (sensitivity −0.99, not shown in Table 1). This analysis revealed that the model was most sensitive to both increases in 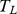 and reductions in 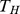. The model also showed a high sensitivity to increases in the parameters maximum surface area-specific assimilation rate, 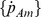 (sensitivity 0.20), volume-specific somatic maintenance cost, 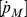 (sensitivity −0.14), and the proportion of energy allocated to somatic maintenance and growth, 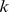 (sensitivity 0.11, Table 1). Changing the parameters half-saturation coefficient, 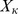, post-metamorphic shape coefficient, 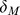, energy conductance, 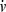, volume-specific cost of structure, 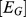, energy investment to transition between life stages (birth 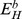, metamorphosis 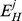, and puberty 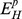), maturity maintenance rate coefficient, 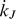, Arrhenius temperature, 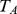, and Arrhenius temperature at upper and lower limits (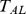 and 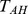) had little effect on growth (sensitivity <0.10). Finally, because the exercise was performed assuming *ad libitum* food supply of a post-metamorphic individual, varying parameters volume-specific cost of maintenance during starvation, 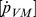, and larval shape coefficient, 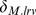, had no effect on the model’s output (Table1).

## Discussion

We satisfactorily parameterized a Dynamic Energy Budget model for the quintessential keystone predator *Pisaster ochraceus*, although independent tests of the model reveal varying estimates of model skill. By combining the theoretical framework of DEB with empirical data collected for modeling purposes, we estimated a set of parameters (Table 1) that describe dynamics of underlying physiological processes related to development, maintenance, growth and reproduction, which in turn define the physiological and ecological performance of *Pisaster* (Figs. 5–8).

### 6.1. Model sensitivity

Future applications of this model should recognize that different parameters have a different relative influence on the model’s output. Thus, depending on users’ specific study objectives, one should consider the precision with which certain parameter values were determined, and whether further tuning is required. Our model sensitivity analysis provided a useful means for assessing this. Those parameters with high sensitivity have a big impact on the output of the model (e.g. thermal sensitivity parameters 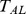 and 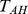), and therefore future efforts should focus on methods for improving their estimation. In contrast, because parameters with low sensitivity should have little influence on the output of the model, their estimation could be treated with less care. Consequently, despite the large variability observed in some of the parameters, their relative importance could be minor if their sensitivity is low (e.g. maturity-maintenance rate coefficient, 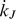).

### 6.2. Reserves and starvation

**Figure 9:**
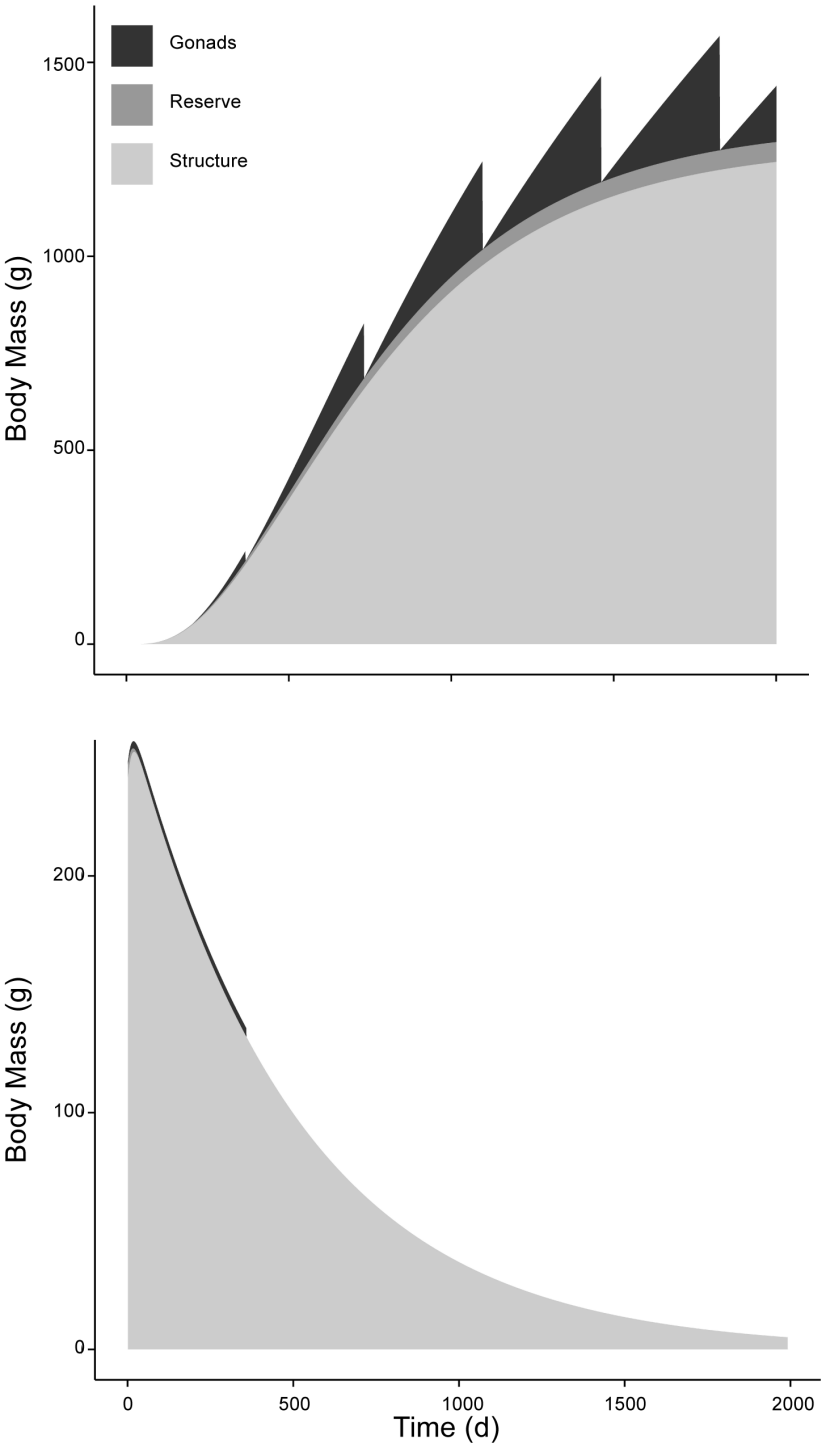
Change in wet weight under abundant food versus starvation. Values are results of DEB simulation using mean parameter values at a temperature of 13°C. Wet weights of gonad (black), reserve (dark grey), and structure (light grey). Panel A is trajectory with food *ad libitum*, and B is trajectory during complete starvation.

The model allows discriminating between the contributions from reserves, structure, and gonads to the total wet weight of an individual experiencing different levels of food availability (Fig. 9). Notably, the contribution of the reserve to the animal’s body mass is very small, albeit enough to fuel its metabolic demands. Similarly, a study conducted with the Atlantic Bluefin Tuna (*Thunnus thynnus*) found a low contribution from reserves (7%) [48] which, according to the authors’ analysis, explains their limited ability to survive starvation and the need to forage voraciously. Despite the even smaller reserve compartment in *Pisaster* (3.8%), its ability to readily draw energy from structure appears as a strategy to cope with naturally uncertain food conditions. The observation that individuals facing food limitation not only show a steady body mass loss but also a reduction in arm length (i.e. structural length) suggests that individuals readily draw energy from the structure compartment to supplement energy allocation from reserves. Now consider a well fed individual (∼250 g wet mass) suddenly deprived of food; the model predicts an exponential decrease in body mass, in accordance with our empirical observations (Fig. 7). Figure 9B illustrates the very short period needed to empty the reserve compartment (∼67 d to reach 1% of the maximum reserve density). Then, as mobilized energy cannot satisfy the maintenance requirements, structure is used as an energy source contributing to the subsequent mass loss. Figure 9 also shows the contribution of gonads to total body mass, which fluctuates annually between 0 and 20.7% in a well-fed individual. Structure, in turn, comprises most of *Pisaster* weight: up to 96.1% (Fig. 9A). Food deprivation further impacts the amount of gonads produced during this initial period, which falls to zero after the annual spawning event (Fig. 9B).

It should also be mentioned that the contribution to total wet weight from the model’s reserve compartment does not reflect the relative contribution from pyloric caecum, which is traditionally regarded as the sea star energy reserve organ [55]–[57], [75]. Although DEB reserves do not account for a large portion of the weight of *Pisaster* (Fig. 9), pyloric caecum is known to reach relative values comparable to reproductive output (∼0.15–0.20 of total body mass) when prey is available *ad libitum* [61]. This seeming contradiction may be explained by the location of the DEB reserve compartment in the energy flow pathway (Fig. 1), which differs from the role of the pyloric caecum in sea stars. Although the pyloric caecum can be considered as an energy storage organ, our assumption is that it is located down-stream from the reserve compartment, in closer proximity to the reproductive buffer. We make this argument based on two lines of evidence. First, DEB theory assumes that when food supply is constant, the DEB reserve density should not vary [11], [22]. The cyclic nature of the pyloric caecum in *Pisaster*, even when prey is available *ad libitum* and individuals’ feeding does not fluctuate [59], [62], [75], conflicts with the idea of equating the DEB reserve compartment with pyloric caecum. Second, studies have shown strong relationships between the volumes of pyloric caecum accumulated during the feeding period of *Pisaster*, and the gonadal tissue produced subsequently during the spawning period [59], [61], [75]. Thus, while it is possible that maintenance is paid in part by pyloric reserves, especially during starvation [56], most of that energy is allocated to gonadal growth. For simplicity, we did not include a pyloric caecum compartment in the model. Future versions of DEB models for *Pisaster* or any other sea star could consider its dynamics explicitly although notably, model results did not appear to be sensitive to its absence. Because the dynamics in pyloric and gonadal indexes are driven by photoperiod regimes, these models would benefit by incorporating photoperiod in their structure.

To better predict changes in size following starvation, specifically when energy diverted to somatic maintenance and growth is not enough to cover the former, we subjected individuals to complete food deprivation and monitored weight-loss over time (Fig. 7). These data allowed us to define and estimate a new parameter, 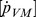, which not only describes energy flows from structure to pay for somatic maintenance, but also provides a good match between observed and simulated reductions in size due to starvation. Although the literature suggests that mobilizing energy from structure to pay for somatic maintenance should be less efficient than from reserves [11], [22], our data revealed a lower value of 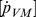 than 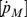 (Table 1). This might be a consequence from the drop in activity and metabolism shown by individuals during prolonged starvation.

Interestingly, animals lost weight smoothly throughout the duration of the starvation experiment (Fig. 7). Previous studies both with vertebrates [76] and invertebrates [54] have shown that the rate of weight loss changes from steep to shallow once reserves are depleted and structure is used as substrate. The observation that reserves make up a small portion of *Pisaster* biomass (Fig. 9) is likely masking the change in rate of weight loss expected based on the literature. Finally, it must be recognized that shrinkage of structure directly translates into a decrease in maintenance costs, consequently allowing the organism to stay alive. This is a key adaptive trait in challenging environments such as the rocky intertidal [35]. Efforts to account for the effect of starvation on organisms that routinely undergo periods of reduced feeding thus represents a crucial step if we are to predict real world dynamics.

### 6.3. Model performance

Because of varying levels of skill amongst different growth metrics, it is important to highlight the instances when the model predictions can be expected to be reliable, and when they should be viewed with caution. The model accurately predicted larval width (Fig. 7) and arm length (Fig. 8A) trajectories. An important strength of DEB is indeed its ability to incorporate the entire life-history of an organism using the same parameter values. Like other species modeled through DEB – including bivalves [77] and fish [48], *Pisaster* undergoes morphological changes between larval and post-metamorphic stages. Accounting for this in the model required application of stage-specific shape coefficients (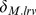, 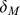) to transform structural lengths to physical lengths and a shape correction function (Eq. 1) to capture growth acceleration. These adjustments provided a good correspondence between real and simulated larval growth. Note that, although the time period covered by the real data is only half of that required for larval competency, the model projection (59.9 d) is close to observations from the literature (∼50 d for well-fed larvae) [70]. While our validation exercise was limited to laboratory conditions with abundant food supply, the feeding functional response embedded in the model structure allows assessments under scenarios of reduced energy availability. If food is limited, the model predicts longer times to larval competency, although maturity level at metamorphosis remains constant. These predictions are consistent with Hart’s [78] study of the urchin *Strongylocentrotus droabachiensis*, and suggest a mechanism for understanding the wide distribution in settlement times previously reported for *Pisaster* (76–228 d) [79]. The model, however, ignores potentially important features of *Pisaster* embryonic and larval developmental stages. For instance, it does not account for the capacity of their larvae to clone when food is abundant and of high quality [70]. Additionally, the model assumes that energy density, 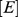, is equal between mothers and offspring, contradicting previous experimental observations revealing that bigger females produced small, low-quality eggs, and small females produced larger, high-quality eggs [32]. Although we disregarded these aspects for simplicity, including them in future versions of the model would certainly increase its potential for bridging the gap between individual and population level processes for *Pisaster*.

Our simulated growth for juveniles and adults also showed good correspondence with empirical data, although precision varied with the size metric considered (predictions for arm length were more precise than for wet weight) (Fig. 8). Several mechanisms may partially explain the reduced precision in predicting wet weight trajectories. First, it is quite common that the weight-at-age data are more scattered than the corresponding length-at-age data, meaning that the former is impossible to capture with the same level of precision as the latter [80]. From a DEB perspective this is not surprising given that weight contains contributions from three state variables (including the structural length) each being a source of the prediction error that adds to the overall amount of the scatter. The physical length, on the other hand, is predicted solely from the structural length, meaning that the corresponding prediction error is the only source of the scatter. Second, precision may be reduced by assuming *ad libitum* food, reserve density remains constant and structural mass increases smoothly with time. Gonadal tissue, however, fluctuates yearly due to spawning events triggered by photoperiodic cycles [59], [75]. By assuming that all mature individuals release their gonads accumulated during the previous year, based entirely on energetic criteria, the model does not capture individual and population level variability in the timing of spawning given by unaccounted potential cues (e.g. body temperature, presence of conspecifics [81], or by photoperiod [59], [75]). Due to the large portion of body mass that can be attributed to gonads during spring-summer period [60], [61], discrepancies in the exact timing of spawning between the model and empirical data can translate into large differences in wet weight at specific times. Note that, when accurate estimates of spawning time are a key modeling goal, reducing the time resolution of the model from days (default) to weeks would improve the value of model’s skill metric; in addition, using a day-length cue for spawning would also improve skill metric. The model’s precision may be even less in case individuals fail to spawn on spring-summer (after accumulating gonads), and/or if the handling time of prey items varies, affecting their capacity to process energy efficiently. Both scenarios are possible under lab and certainly field conditions [34].

An additional source of error when modeling wet weight trajectories may come from the observation that relative investment in gonads negatively correlates with food availability across sites in *Pisaster* [61], which deviates from DEB theory’s assumption that the relative investment (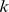) is constant. Sanford and Menge [61] hypothesized that such an adaptation may increase the likelihood for larvae produced at poor sites to reach worthier locations. For simplicity, and because the mechanism is not completely understood, our model ignores this hypothesis.

Because of the ecological importance of the age at puberty, it is worth touching on the large discrepancy between the modeled and observed values (264 d and 5 y, respectively). Two aspects may be determining the mismatch. First, the observed value is an estimate calculated using field observations [71], where environmental conditions (notably food and temperature) are uncertain and individuals probably do not forage constantly. In contrast, our estimate is based on growth measurements collected in controlled, constant lab settings, where *Pisaster* could feed *ad libitum*. Second, the difference between observed and modeled age at puberty may be due to the uncertainty in the estimates of some of the DEB parameter values. For example, our estimate for maturity maintenance rate coefficient was 0.0000029±0.018 (mean±SD) (Table 1).

### 6.4. Environmental dependency

Throughout its wide geographic range, *Pisaster* often copes with extremely challenging conditions inherent to the rocky intertidal. Stress may arise from both physical and biological forces whose impacts vary spatially and temporally. Here we focused on body temperature and food availability because of their overarching influence on physiological and ecological performance [80]. First, our thermal sensitivity experiment yielded a complete thermal performance curve for respiration rate (hereafter, TPC) for *Pisaster* (Fig. 3). A number of different approaches have been proposed to analytically characterize TPCs ([e.g. [82], [83]), most of which typically arrive at the same general shape; namely, an increase in performance with temperature, followed by a leveling off at an intermediate temperature (optimal performance), and a subsequent drop leading to minimum performance and death at extreme temperatures [84]. The five parameters we estimated here determine this general shape. TPCs are becoming an increasingly popular tool to readily assess the effect of temperature on relevant ecological and physiological performance traits, as well as for predicting impacts of climate change [2], [84]. When used in a DEB framework, one can further discriminate among the effects of temperature on the various physiological processes being modeled (maturity, maintenance, growth, reproduction). Since the relative importance of these processes may vary depending on the organism’s maturity (e.g. reproduction is only a defining trait after maturity has been reached), being able to quantify their responses to temperature separately should prove useful when working across life-stages. Note, however, that our thermal sensitivity parameters were estimated based on oxygen consumption measurements, and we rely on the assumption that all physiological rates respond to temperature following the same formulation. While empirical evidence sustains this assumption [11], we recommend testing it against independent measurements of feeding or growth rates at a range of temperatures, particularly at extreme ends of the curve, where different processes are expectedly less coupled [15], [85]. In addition, our model assumes that temperature exerts the same effect on metabolism, regardless of whether individuals are aerially exposed at low tide or submerged at high tide. We based this on a recent study conducted on *Pisaster*, which showed that thermal sensitivity is virtually equal between submerged and exposed animals subjected to a range of temperatures; Q_10_ values being 2.18 and 2.12, respectively [86]. However, despite finding similar sensitivities, the study also revealed a significant reduction in oxygen consumption rates (metabolic depression) for sea stars exposed to air compared to those kept submerged in water at the same temperatures [86]. The mechanism by which some intertidal organisms reduce metabolism during aerial exposure is unclear, and therefore we did not consider it in the model. Note, however, that if animals are exposed daily, cumulative metabolic depressions may potentially have important consequences for long-term energy budgets. It should also be pointed that, since our TPC was described based on aquatic conditions, our model may not work when body temperature during aerial exposure exceeds the peak of our curve (∼295 K, or 22°C). Since aerial body temperatures above that threshold are known to occur for *Pisaster* [86], [87], models employed to describe its condition during periods of aerial exposure should add an additional set of thermal sensitivity parameters. While the value for Arrhenius temperature (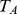) would not change, the parameters that define the curve’s shape at extreme temperatures (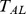, *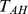,* 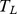 and 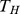) should be re-estimated based, for example, on information of critical temperatures [87]. Finally, temperature sensitivity parameters are likely to vary as a function of both prevalent body temperatures at the collecting sites/intertidal height (i.e. acclimatization) and details related to experimental design (e.g. acclimation time; chronic vs. acute) [87]. Future studies must therefore carefully consider these and other caveats reported elsewhere [88], in order to avoid misinterpreting modeling results.

Moreover, our feeding experiment yielded a scaled Type II functional response curve (Fig. 2) which, based on a half-saturation coefficient, 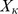, provides means for assessing the effect of changing food density on the rate of energy intake [11]. To our knowledge, this curve had not been described for *Pisaster* before.

### 6.5. Conclusions

In a period of increasing anthropogenic pressure, anticipating changes in the dynamics of ecological systems represents a complex, yet necessary challenge that ecologists must face in order to prevent further collapses of natural resources [4]. Difficulties arise, in part, as a result of the multiple processes taking place across levels of biological organization, which appear linked to nonlinearities emerging at broad scales [3]. Predicting dynamics of complex systems requires first uncovering the mechanisms behind such nonlinearities [1], and then their incorporation in a coherent modeling framework [22]. By blending the virtues of experimental and theoretical biology [24], recent advances are providing increasingly accurate predictions of interdependent physiological and ecological processes occurring simultaneously, thus advancing our understanding of emergent properties that would otherwise remain obscure.

The DEB model presented here represents a step forward in our efforts to bring data and theory together, to help illuminate key physiological properties and their dependence on biotic and abiotic environmental drivers. Given the keystone role of *Pisaster* [89], [90], insights obtained from this individual-based mechanistic model can potentially shed light on dynamics at population and community levels [15], [87], especially when comparable models are developed for other ecologically key players in the intertidal ecosystem.

## Supporting Information

Appendix S1 General description of a Dynamic Energy Budget model for a standard organism.
(DOCX)
