# Supplementary material for: A Dynamic Energy Budget (DEB) Model for the Keystone Predator Pisaster ochraceus
Source: PLoS One. 2014 Aug 28;9(8):e104658. doi: 10.1371/journal.pone.0104658 (PMC4148243; doi:10.1371/journal.pone.0104658)
Supplement: Appendix S1 — General description of a Dynamic Energy Budget model for a standard organism. (DOCX) [file pone.0104658.s001.docx]

**Appendix S1**

**Generalized DEB model structure**

This section will describe some basic features of a standard DEB model (for deeper discussions of the fundamentals behind the theory see [[1](#_ENREF_1),[2](#_ENREF_2)]). Standard versions of DEB models conceptually discriminate between the state variables energy reserve, $E$ (J), structural volume, $V$ (cm^3^), and maturation, $E_{H}$ (J). Once the threshold of puberty has been reached, the state variable reproductive buffer, $E_{R}$ (J), can be included. Reproductive buffer accounts for variability in the reproductive potential of mature individuals. The mass of an organism at any given point in time is defined by the contributions from reserve, structure, and reproductive buffer. Maturation, in turn, is understood as energy or mass that dissipates in the form of heat or metabolites as the organism increases its maturity; therefore, this state variable does not contribute to total mass. A chief assumption in standard DEB models is that the biochemical composition of reserve and structure are constant (i.e. strong homeostasis assumption). Although the state variables cannot be measured directly, their dynamics are fully described by a set of equations that will ultimately characterize an organism’s physiological condition [[3](#_ENREF_3)].

Before defining the processes that govern an individual’s physiological condition, it is worth elaborating on how DEB theory deals with matters of size and shape. Assuming that the organism’s shape does not change with growth (i.e. isomorphy), the model relies on structural length $L$ (cm), rather than physical length $L_{W}$ (cm), to provide a measure of size. Structural length is preferred because (1) it only relates to structural volume discriminating between contributions from other state variables, and (2) it is not affected by the organism’s shape, thus favoring inter-species comparisons [[1](#_ENREF_1)]. The DEB parameter shape coefficient $\delta_{M}$ (dimensionless) serves to translate physical measurements taken from some representative length (e.g. arm length) to structural length: $L=\delta_{M}\cdot L_{W}$. In the model, structural length defines all physiological processes proportional to area or volume. The equations describing surface-area related processes are expressed in terms of $L^{2}$ (cm^2^), while those proportional to volume are expressed in terms of $L^{3}$ (cm^3^). All rates (units t^-1^) are written with a dot as in . All surface-area specific quantities (units L^-2^) are written in curly braces as in . All volume-specific quantities (units L^-3^) are written in square brackets as in .

Energy reserve changes as the organism acquires food. DEB theory makes use of a scaled version of Holling’s type II functional response model [[4](#_ENREF_4)], *f* (dimensionless), to account for the effects of food availability, *X* (resource density, 2-cm shell length mussels m^-2^), on feeding and assimilation flux. The amount of energy entering the body is assumed to be proportional to the surface-area of the structural volume, i.e. $L^{2}$ (cm^2^). Thus, as the organism forages the energy assimilated through the gut, (J d^-1^), can be described by:

with $f=\frac{X}{X+X_{\kappa}}$ ,

where is a DEB parameter known as maximum surface area-specific assimilation rate (J d^-1^ cm^-2^) and $M$ is a shape correction function (dimensionless) explained in the main text (Eq. 1). The parameter $X_{\kappa}$ represents the half-saturation coefficient or Michaellis-Menten constant (resource density at which feeding rate is one half of its maximum value) [[5](#_ENREF_5)]. The process of assimilation is not perfect; inefficiencies in transforming energy from food into energy reserve determine that a fraction of the available energy is dissipated.

The energy stored as reserve is balanced by all the metabolic needs of the organism, including growth, development (i.e. maturity), reproduction and maintenance (structural and maturity) [[6](#_ENREF_6)], as well as by the energy dissipated through the processes of growth and reproduction. The total energy allocated for those needs is known as utilization flux, (J d^-1^). Both the assimilation and the utilization fluxes define the dynamics of the reserve $E$:

,

where three DEB parameters are introduced; energy conductance, (cm d^-1^), volume-specific cost of structure, $\left[E_{G}\right]$ (J cm^-3^), and $\kappa$ (dimensionless, explained below). The equation for estimating has been derived assuming that reserve density, $\left[E\right]=E/V$ (J cm^-3^), follows first order dynamics – i.e. the rate of decrease of reserve density is proportional to the amount of reserve density [[7](#_ENREF_7)]. Notably, this aspect of DEB theory offers a mechanism for filtering the effects of highly variable environmental conditions, thus suiting the organism with a homeostatic capacity. In depth explanations of the formal derivation of can be found in Kooijman [[1](#_ENREF_1)] and Jusup et al. [[8](#_ENREF_8)].

The utilized energy is then distributed among the metabolic processes – somatic maintenance, (J d^-1^), structural growth, (J d^-1^), maturity maintenance, (J d^-1^), and maturation or reproductive buffer, (J d^-1^) (Fig. 1). The long-standing problem of allocation has been solved by DEB theory via the so-called *kappa* ($\kappa$) rule [[1](#_ENREF_1),[9](#_ENREF_9)]. The parameter $\kappa$ amounts to a fixed fraction of energy utilized from the reserves that goes to somatic maintenance and growth, the former having absolute priority over the latter. For ectothermic organisms, somatic maintenance amounts to the energetic costs associated with the turnover of structural proteins and the maintenance of metabolite concentration gradients across cell membranes. Since all these costs are proportional to structural volume, somatic maintenance can be described by:

,

where is a parameter known as volume-specific somatic maintenance cost (J d^-1^ cm^-3^). Due to the priority given to somatic maintenance, the energy derived to structural growth can be calculated from . Growth is understood as a change in structure (excluding dynamics in body size due to fluctuations in energy reserve and reproductive buffer), which can be described by [[8](#_ENREF_8)]:
 .

Note that equation 5 includes the parameter volume-specific cost of structure $\left[E_{G}\right]$ to account for the cost of converting energy from reserve to structure (including tissue production and anabolic overheads). This formulation is equivalent to the traditional von Bertalanffy growth equation [[10](#_ENREF_10)], whose parameter von Bertalanffy growth coefficient, (d^-1^) describes the decreasing rate at which individuals reach their ultimate size $L_{\infty}$ resulting from the balance between food assimilation and somatic maintenance [[6](#_ENREF_6),[7](#_ENREF_7)]. Furthermore, this mechanism is incorporated in DEB theory’s formulation for this parameter; . The validity of this formulation has been confirmed by successfully modeling the growth trajectories of many taxa reported in the literature [[see 1 for details](#_ENREF_1)].

The utilized energy not going to somatic maintenance and growth, , is channeled to cover costs of maturity maintenance, , and either increase the level of maturity or fill up the reproductive buffer, ; energy allocated to maturation is assumed to increase from the age at birth until puberty, after which the available energy is directly used for building-up the reproductive buffer (Fig. 1). Maturity maintenance, (J d^-1^), which accounts for the maintenance of increased complexity attained throughout development, is assumed proportional to the level of maturity and can be modeled by:

,

where the parameter represents the maturity maintenance rate coefficient (d^-1^). Once puberty is reached ($E_{H}\geq E_{H}^{p}$), maturity maintenance becomes constant. Knowing the energy allocated to maturity maintenance, the dynamics of can be tracked through:

.

While is equivalent to the rate of change of the maturation state variable (i.e.$dE_{H}/dt$) before puberty, it describes dynamics of the reproductive buffer state variable (i.e. $dE_{R}/dt$) after puberty is reached. Gonadal tissue is then synthetized from the reproductive buffer. The efficiency of turning reserve energy into eggs or sperm is determined by a reproductive efficiency coefficient $k_{R}$. We refer to the maturation state variable to determine the level of maturity at any given point in time, as well as the timing of transitions between developmental stages. Explicitly relying on the state variable maturation liberates the model from having to use size as a metric for developmental stage. This feature is particularly relevant for species that can grow or shrink indeterminately, such as sea stars (Feder, 1956; Sebens, 1987).

Physiological rates are temperature-dependent, and need to be corrected accordingly. DEB models make use of the Arrhenius relationship to describe the influence of body temperature on physiological rates over the range of temperatures where enzymes can be assumed to be active, delimited by the parameters $T_{L}$ (K) and $T_{H}$ (K). The parameter $T_{A}$, known as Arrhenius Temperature, allows capturing the thermal-sensitivity of the organism within these margins. Above and below the thermo-tolerance window enzymes become inactive, leading to a decline in physiological rates, which can be traced by the parameters $T_{AL}$ and $T_{AH}$, respectively [[11](#_ENREF_11),[12](#_ENREF_12)]. These five parameters fully define an organism’s thermal performance curve, in accordance to the formula:

,

where is the value of the physiological rate at a given body temperature $T$ (K), and is the known value at a reference temperature $T_{1}$ (K).

Finally, DEB models explicitly acknowledge the existence of overhead costs associated with processes where energy-conversion inefficiencies between different compartments are observed. Such overhead costs, linked to assimilation, growth, and reproduction (Fig. 1), translate to energy losses in the form of heat and metabolites [[1](#_ENREF_1)].

**Appendix S1 References**

1. Kooijman SALM (2010) Dynamic Energy Budget Theory For Metabolic Organization. Cambridge: Cambridge University Press. 490 p. p.

2. Kooijman SALM, Sousa T, Pecquerie L, van der Meer J, Jager T (2008) From food-dependent statistics to metabolic parameters, a practical guide to the use of dynamic energy budget theory. Biological Reviews 83: 533-552.

3. Sousa T, Domingos T, Kooijman SALM (2008) From empirical patterns to theory: a formal metabolic theory of life. Philosophical Transactions of the Royal Society B: Biological Sciences 363: 2453-2464.

4. Holling CS (1959) Some characteristics of simple types of predation and parasitism. Canadian entomologist 91: 385-398.

5. Saraiva S, van der Meer J, Kooijman SALM, Sousa T (2011) Modelling feeding processes in bivalves: A mechanistic approach. Ecological Modelling 222: 514-523.

6. Sousa T, Domingos T, Poggiale J-C, Kooijman SALM (2010) Dynamic energy budget theory restores coherence in biology. Philosophical Transactions of the Royal Society B: Biological Sciences 365: 3413-3428.

7. van der Meer J (2006) An introduction to Dynamic Energy Budget (DEB) models with special emphasis on parameter estimation. Journal of Sea Research 56: 85-102.

8. Jusup M, Klanjscek T, Matsuda H, Kooijman SALM (2011) A Full Lifecycle Bioenergetic Model for Bluefin Tuna. PLoS ONE 6: e21903.

9. Kooijman SALM (1986) Energy Budgets Can Explain Body Size Relations. Journal of Theoretical Biology 121: 269-282.

10. Von Bertalanffy L (1957) Quantitative laws in metabolism and growth. The Quarterly Review of Biology 32: 217-231.

11. Freitas V, Campos J, Fonds M, Van der Veer HW (2007) Potential impact of temperature change on epibenthic predator-bivalve prey interactions in temperate estuaries. Journal of Thermal Biology 32: 328-340.

12. Sharpe PJH, DeMichele DW (1977) Reaction kinetics of poikilotherm development. Journal of Theoretical Biology 64: 649-670.
